# Comorbid somatic conditions among older patients with major mental illness: A retrospective inpatients study.

**DOI:** 10.1192/j.eurpsy.2023.1989

**Published:** 2023-07-19

**Authors:** M. Karoui, A. Maaroufi, N. Houssem, R. Kammoun, F. Ellouze

**Affiliations:** 1 Psychiatrie, Faculté de médecine de Tunis, Tunis, Tunisia

## Abstract

**Introduction:**

Psychiatric admissions of elderly subjects with mental disorders is a difficulty in management and treatment. This problem is essentially related to somatic comorbidities of this population.

**Objectives:**

To compare the demographic characteristics, comorbid conditions and functional status of younger and older individuals with major mental illnesses admitted to psychiatry.

**Methods:**

Using the records of patients admitted between 2015 and 2020 to the psychiatric department "G" of Razi Hospital, we collected the demographic characteristics, comorbid conditions and functional impairments for patients admitted to psychiatry and aged over 65 years. We compared these characteristics to those collected in the total population of admitted patients.

**Results:**

The study population consisted of 75 elderly patients. Compared to the youngest patients there was a greater male majority, a higher rate of severe cognitive impairment, higher rates of chronic medical illnesses, such as congestive heart failure.

**Conclusions:**

Our findings suggest an urgent need for integrated psychiatric and medical care for elderly subjects with major mental illness. Future research is needed to adapt evidence-based integrated models of collaborative mental health care.

**Disclosure of Interest:**

None Declared

